# Machine Learning and Radiomic Features to Predict Overall Survival Time for Glioblastoma Patients

**DOI:** 10.3390/jpm11121336

**Published:** 2021-12-09

**Authors:** Lina Chato, Shahram Latifi

**Affiliations:** Department of Electrical and Computer Engineering, Howard R. Hughes College of Engineering, University of Nevada, Las Vegas (UNLV), Las Vegas, NV 89154, USA; shahram.latifi@unlv.edu

**Keywords:** high grade glioma, brain tumor, magnetic resonance imaging, machine learning, edema, enhanced tumor, tumor core, neural network, accuracy

## Abstract

Glioblastoma is an aggressive brain tumor with a low survival rate. Understanding tumor behavior by predicting prognosis outcomes is a crucial factor in deciding a proper treatment plan. In this paper, an automatic overall survival time prediction system (OST) for glioblastoma patients is developed on the basis of radiomic features and machine learning (ML). This system is designed to predict prognosis outcomes by classifying a glioblastoma patient into one of three survival groups: short-term, mid-term, and long-term. To develop the prediction system, a medical dataset based on imaging information from magnetic resonance imaging (MRI) and non-imaging information is used. A novel radiomic feature extraction method is proposed and developed on the basis of volumetric and location information of brain tumor subregions extracted from MRI scans. This method is based on calculating the volumetric features from two brain sub-volumes obtained from the whole brain volume in MRI images using brain sectional planes (sagittal, coronal, and horizontal). Many experiments are conducted on the basis of various ML methods and combinations of feature extraction methods to develop the best OST system. In addition, the feature fusions of both radiomic and non-imaging features are examined to improve the accuracy of the prediction system. The best performance was achieved by the neural network and feature fusions.

## 1. Introduction

Gliomas are common brain tumors that start in the glial cells, which are gluey supportive cells that surround nerve cells in the brain, and they represent 80% of primary malignant brain tumors [1,2]. These tumors can affect brain function and be life-threating depending on growth rate, size, and location. Glioma tumors are classified into two types according to their aggressiveness: low-grade glioma (LGG) and high-grade glioma (HGG) [3,4]. Some LGGs are benign tumors [5], while HGGs are malignant tumors [6]. Malignant tumors are aggressive tumors that contain cancerous cells and are life-threatening. These tumors have irregular boundaries with a high growth rate. Glioblastoma multiforme (GBM) tumors are the most common type of HGG [3,7,8], classified as a grade IV glioma, the most aggressive grade of brain tumors, by the World Health Organization (WHO) [2,9,10]. These tumors represent 50% of gliomas [2,11,12]. Furthermore, 90% of GBMs are primary tumors [9,13]. Unfortunately, patients with GBM tumors have a very poor survival rate and prognosis outcome. Several risk factors are recognized to increase the chance of developing brain tumors. Some of these factors can be controlled and are related to the patient’s lifestyle behaviors, such as smoking, dietary habits, and alcohol intake. However, other factors cannot be controlled such as age, family history, and genetics. In addition, some diagnosis methods that are based on ionizing radiation, such as prenatal diagnostic X-ray exposure, increase the risk of developing childhood brain tumors [14,15]. According to the American Cancer Society (ACS), the GBM patient’s age is associated with the survival rate, which is better for young people than for old people [16,17,18]. For example, the 5 year relative survival rate is 22% for patients aged 20–44 years, whereas it is 6% for patients aged 55–64 years [17]. To diagnose glioma brain tumors accurately, different tests are required, such as neurological exams, imaging tests, and biopsy tests [19,20,21]. Due to the superior soft tissue contrast of magnetic resonance imaging (MRI) scans, which allow for better visualization of the complexity and the heterogeneity of tumor regions, it is recognized as a gold imaging method to identify and localize brain tumors [20,22]. Glioma brain tumors have a variable prognosis and various heterogeneous histological subregions, which are reflected in their imaging phenotype [23,24,25]. In general, the common brain tumor subregions are peritumoral edema, necrosis, cyst, and enhancement tumor [26]. Different structural MRI modalities, such as T1-weighted (T1) scans, T2-weighted (T2) scans, contrast-enhanced T1-weighted (cT1) scans, and fluid-attenuated inversion recovery (FLAIR) scans, can be used to visualize different brain tumor subregions [22]. For example, T2 and FLAIR scans visualize the peritumoral edema as a bright region [26,27]. Necrosis is identified as a bright region in the T2 scan but a dark region in the T1 scan, with an irregular enhancing border in the cT1 scan [26]. The cyst is recognized as a dark, rounded region in the T1 scan and is a very bright region in the T2 scan [26]. Another study used cT1 scans to justify the existence of the cystic component [28]. However, the existence of a cyst is rare as GBM is commonly developed as a unilateral solid tumor [28]. The enhancement tumor (ET) region is defined as a bright region surrounding the cystic/necrotic components, and the Multimodal Brain Tumor Segmentation challenge (BraTS) recognized it by comparing T1 and cT1 scans [29]. The tumor core (TC), which includes both the ET region and cystic/necrotic components, can be visible in the T2 scan, while the whole tumor (WT) region is visible in the FLAIR scan [29]. 

To improve the poor prognosis of GBM, various clinical investigations studied the connection among appearance, size, and location of GBM tumor subregions in relation to the poor survival rate [30,31,32]. Tissue death in cancers is called necrosis, and it was found that the existence of a necrotic tumor is associated with a poor survival rate [33]. Peritumoral edema can be defined as a characteristic feature of malignant glioma regarding the extent of neovascularization and vascular endothelial growth factor (VEGF) expression [30,34]. Angiogenic and vascular permeability factors associated with infiltrating tumors are identified as reasons for edema development [35,36], which is often presented in GBM and associated with poor prognosis [27,37]. Wu et al. found a shorter survival rate in glioblastoma patients due to the existence of both edema and necrosis [26]. Qin et al. summarized that the surgical treatment of edema delayed postoperative recurrence and relapse rates [27]. In addition, the survival rate of glioma patients is associated positively with the appearance of cystic tumor components [32]. 

Due to the power of ML methods in developing accurate prediction systems that can identify complex and nonlinear patterns in different data types, they have recently been used to improve the healthcare systems in enhancing the diagnosis process and drug discovery [38,39], as well as in deciding a suitable treatment plan [40,41,42,43]. The term ML represents all traditional ML methods, such as the support vector machine (SVM), neural network (NN), trees, random forest (RF), and K-nearest neighbor (KNN), including deep learning (DL) methods, which are just NNs with a very deep structure. Recently, most researchers have used the term “ML” for traditional ML methods and “DL” for deep neural network methods. Various studies have involved prediction methods based on ML/DL and medical data to predict prognosis outcomes. Different types of medical images were used to develop an OST prediction system for different cancers [44,45,46,47,48,49]. For example, MRI images were used for glioma brain tumors and HGG tumors [45,48], while CT scans were used for pancreatic ductal adenocarcinoma, head and neck squamous cell carcinoma, lung, and gallbladder cancers [44,46,47,49].

To develop state-of-the-art OST prediction methods for GBM patients, the BraTS challenge provided well-processed medical data based on medical imaging information (MRI) and non-imaging information [23,29]. Numerous methods based on ML and radiomic features were proposed and examined [50,51,52,53,54,55], and details about the top ranked methods are listed in Table 1 for BraTS 2018 and 2019. The BraTS validation and test datasets are unlabeled data as described in [56]. The validation data were provided to evaluate developed prediction models and to choose the best model for the test phase. The test data were provided to test and evaluate the final (best) prediction model. The participant should conduct predictions and submit the results to the BraTS challenge for evaluation. From Table 1, it is obvious that tumor size, which represents the volume information of tumor/tumor subregions, is an important radiomic feature in developing an automatic prediction model for GBM patients. 

In this paper, we propose a novel method to extract volumetric and location information of GBM tumors, as well as the tumor subregions. Our proposed feature extraction method is based on calculating the volume of the GBM brain tumor and the tumor subregions in different brain function regions. To our knowledge, there is no automatic software/program that identifies each brain functional region (i.e., lobe) in structural MRI scans. Therefore, an alternative method is proposed to divide a brain volume into two sub-volumes (regions) using the brain section planes (mid-sagittal, mid-coronal, and mid-horizontal). Then, the volumes of the tumor region and subregions are calculated in each brain sub-volume. As the three brain section planes are used, three approaches are proposed to extract three different sets of radiomic features to train an ML prediction system. The BraTS 2019 dataset was used to develop our prediction system based on a classification process, which classifies a GBM patient into one of three survival groups: short-term, mid-term, and long-term.

The remainder of this paper is organized as follows: Section 2 presents the methods and materials used to develop and evaluate the OST prediction system for GBM patients; Section 3 lists and illustrates experiments and results that were used to test and evaluate the proposed prediction system; Section 4 discusses the achieved results and compares them with previous studies; Section 5 concludes this work and highlights future work to improve the performance of the proposed prediction system. 

## 2. Materials and Methods

**Figure 1:**
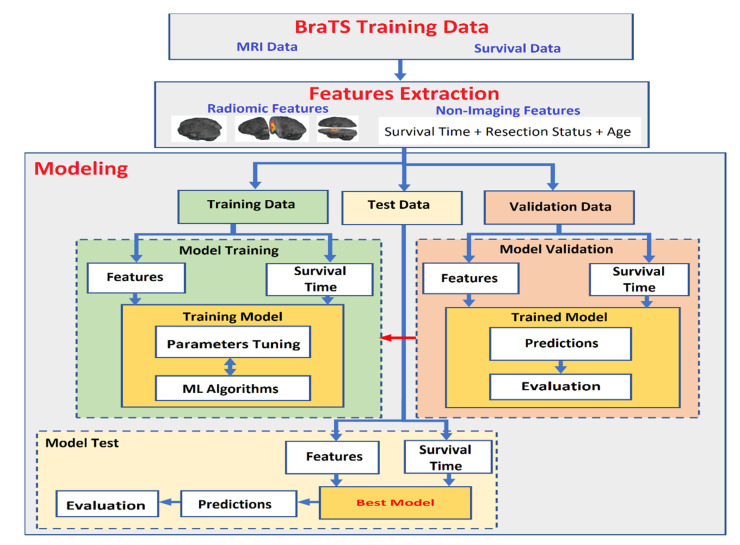
Steps to develop the overall survival time prediction system for glioblastoma patients.

In this paper, medical data based on medical imaging information and non-imaging information were used to develop an accurate OST prediction system for GBM patients. Therefore, traditional ML methods were used to train the OST prediction system. Most medical images are 3D and have a big size in terms of the number of voxels. Sometimes they are not suitable to train reliable prediction models based on traditional ML methods when the size of the dataset is small. Thus, a proper feature extraction method is used to derive meaningful descriptors from medical images to train an accurate prediction model. To develop an automatic OST prediction system for glioblastoma patients based on a multiclass classification task, two steps are required, as shown in Figure 1. These steps are feature extraction and modeling. The features extraction step is used to develop high-level descriptors from a dataset to produce robust features. The second step is modeling that is used to train, test, and evaluate the prediction system using the extracted robust features and an ML algorithm. 

### 2.1. Dataset

**Figure 2:**
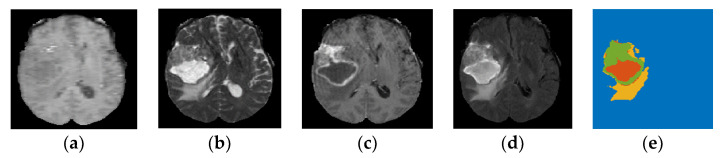
Multimodal MRI images based on 2D representation with segmentation labels (yellow: Ed, green: ET, red: NCR/NET) for a sample from the BraTS 2019 dataset-HGG group. (**a**) T1; (**b**) T2; (**c**) T1 Gd; (**d**) FLAIR; (**e**) labels.

**Figure 3:**
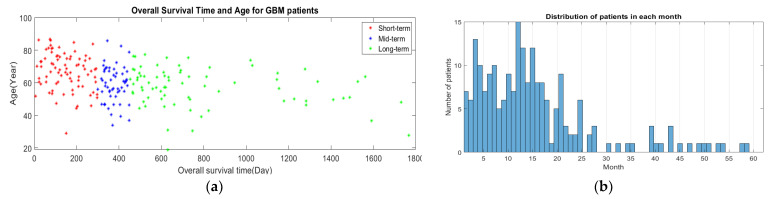
Survival distribution for BraTS 2019 dataset, (**a**) patients’ age and the overall survival time, (**b**) number of patients in each month.

**Figure 4:**
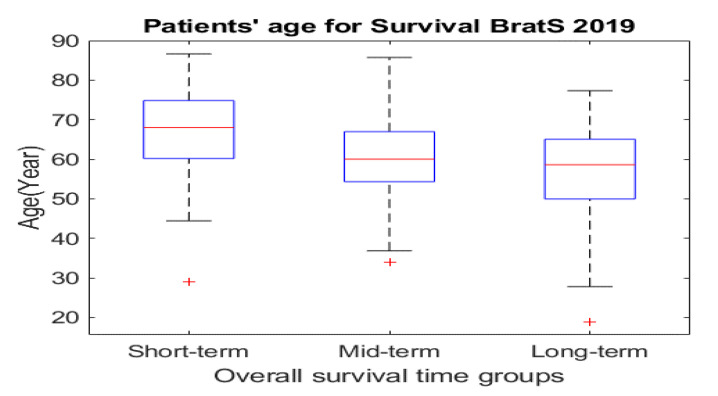
Boxplot of patients’ age for the three survival groups.

BraTS 2019 released well-processed medical data to segment glioma brain tumors automatically from medical images for HGG and LGG patients, as well as to predict the OST for HGG patients [23,29]. These medical data consist of medical imaging data and non-imaging data. As presented in Section 1, different modalities are used to accurately visualize each brain tumor subregion. Thus, the BraTS challenge provided four MRI scans (T1, T2, gadolinium contrast T1 (T1 Gd), and FLAIR) for each HGG and LGG patient, as they used them to annotate brain tumor subregions. The non-imaging data contained the patient’s age in years, survival time in days, and resection status for HGG patients only. These data contained three categories of resection status: gross total resection (GTR), subtotal resection (STR), and unknown resection status. The BraTS challenge provided a labeled imaging training dataset by professional radiologists for segmenting glioma subregions. The medical imaging data constituted 335 samples (i.e., patients), and each sample had 3D multimodal MRI scans with a segmentation labels file. Each MRI scan and segmentation file consisted of 155 slices, and the size of each slice was 240 × 240 pixels. The HGG group consisted of 212 samples (patients), with each sample having non-imaging data, as well as medical imaging data. The segmentation labels file consisted of four labels, each representing a specific brain tumor subregion, as well as heathy brain tissues and an image background: Label 1 represents the necrotic and non-enhancing tumor core (NCR/NET) region; Label 2 represents the ED region; Label 4 represents the ET region; Label 0 represents the healthy brain tissues and the background of an MRI image. Figure 2 shows the four MRI modalities with segmentation labels. Detailed information about the survival data is listed in Table 2. The distribution of the patients’ age with respect to the overall survival time of the three survival groups is shown in Figure 3a, and the boxplot of the patients’ age for each survival group is shown in Figure 4.

### 2.2. Feature Extraction Methods

#### 2.2.1. Radiomic Features 

**Figure 5:**
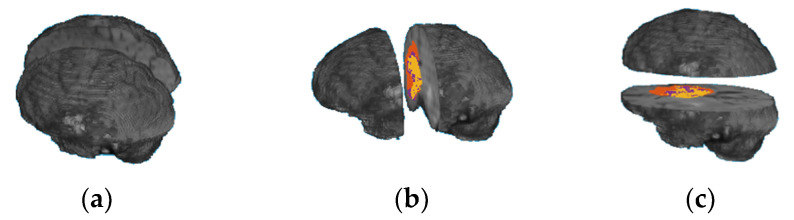
Two brain sub-volumes using the three section plans: (**a**) mid-sagittal; (**b**) mid-coronal; (**c**) mid-horizontal.

A human’s brain and body are divided into two sides, right and left, using the mid-sagittal plane. Each body part is controlled by the opposite part of the brain; for example, the left part of the brain controls the right side of a human body and vice versa. Each brain part contains four main functional regions (lobes): frontal lobe, occipital lobe, parietal lobe, and temporal lobe. In addition, there are other regions of the brain, called the cerebellum and the brain stem. Each of these functional regions is associated with different functions. The main idea of this paper was to develop an OST based on ML methods and volumes of a whole tumor region, as well as the tumor subregions extracted from each brain functionality region (lobe). However, we did not find a software program that could automatically identify each brain functionality region accurately in MRI scans. Therefore, we propose a novel alternative method based on dividing a brain volume into two sub-volumes, using brain sectional planes to calculate volumetric features in each brain sub-volume. Three different approaches are used to divide a brain volume in an MRI scan into two sub-volumes using one of the three brain functional planes, as shown in Figure 5. The first approach is based on using the mid-sagittal sectional plane to divide the brain volume in an MRI image into left and right volumes; the second approach is based on using mid-coronal sectional plane to divide the brain volume into anterior and posterior volumes; and the third approach is based on using the mid-horizontal plane to divide the brain volume into superior and inferior volumes. Twelve volumetric features are calculated from each approach. Five features are extracted from each brain sub-volume, representing the volume of the brain region (Vb), the volume of the whole tumor (Vwt), the volume of the gadolinium (GD)-enhanced tumor, the volume of the NCR/NET tumor, and the volume of the edema. Another two features are extracted from the whole brain volume, representing the volume of the whole brain region and the volume of the whole tumor region. Table A1, Table A2, Table A3 list the 12 features extracted from the mid-sagittal, the mid-coronal, and the mid-horizontal approaches, respectively. The order of the features based on each brain volume is used to describe the location information. All the MRI scans were well normalized, processed, and resampled by the BraTS challenge, and the segmentation labels are provided for this well-processed data. Thus, the number of voxels in any brain tumor subregion represents the volume of that region. To calculate the volume of the whole brain in an MRI scan, the OTSU thresholding method is applied to extract the brain region in an MRI scan, and then the number of voxels for the extracted region is calculated.

#### 2.2.2. Clinical Non-Imaging Features 

The clinical non-imaging information includes the patients’ survival time in days, age in years, and resection status. The survival time represents the target (label) to train the prediction system. The age and resection status are used as non-imaging features. The age is included directly in the feature vector. Two values are used to implement the resection status feature: “1” is for GTR, and “0” is for both STR and unknown resection status.

### 2.3. Modeling OST Classifier

To develop an automatic prediction system that classifies a GBM patient into one of the three survival groups (short-term, mid-term, long-term), ML algorithms were used. The short-term survivor group represents patients with a survival time of less than 10 months; the mid-term survivor group represents patients with a survival time of 10–15 months; the long-term survivor group represents patients with a survival time of more than 15 months. 

To develop an accurate automatic prediction model based on the classification task, a modeling process was required, which included three main steps, as shown in Figure 1: model training, model validation, and model testing. Therefore, the dataset was divided into three sets: training, validation, and testing. The training dataset was the largest in size compared to the validation and test dataset, and it was used to train the model by implementing ML algorithms to predict the outcomes (overall survival time) on the basis of the labeled target. The validation data were used to tune model parameters, checking for any model bias/overfitting problems. The testing dataset were unseen data used to evaluate and test the performance of a prediction model. In the model training step, the extracted features from the training dataset with labels (three survival time groups) were used with ML algorithms to train the prediction model using a supervised learning approach. To produce a reliable prediction model, parameter tuning was required in the model validation step using the validation dataset. This step is very important to avoid model overfitting, as well as to check for any model bias problems. To justify the model’s performance, the model was examined using the testing dataset. To produce a reliable ML prediction model, the k-fold cross-validation method was used to avoid/reduce model overfitting.

## 3. Results

To develop the best OST prediction model based on the proposed radiomic features and ML methods, three experiments were conducted, as described below.

In the first experiment, three sets of radiomic features (mid-sagittal, mid-coronal, and mid-horizontal were extracted from the BraTS 2019 training dataset to train an ML model using a three-class classification process. Six ML methods (NN, SVM, tree, naïve Bayes, linear discriminant, and KNN) were used to develop the OST classification model for each of the three sets of the radiomic features. Several configurations were implemented to produce the best prediction model for each ML method. The best models are listed in Table 3, Table 4 and Table 5 for the mid-sagittal approach, mid-coronal approach, and mid-horizontal approach, respectively. The results show that the best performance was achieved by NN for the three sets of the radiomic features. 

**Figure 6:**
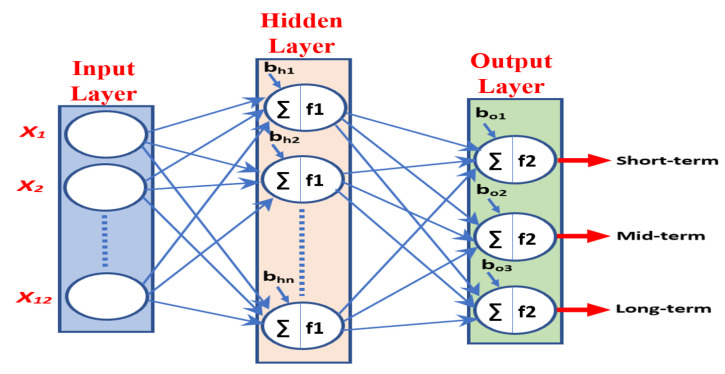
Simple neural network. f1 is tanh, and f2 is softmax.

In the second experiment, the survival rate of GBM patients was associated with the patients’ age, which is better for young people than for old people according to the ACS [16,17,18]. Thus, the age factor was used as a non-imaging feature and was combined with the radiomic features to improve the performance of the OST classification model based on NN. A simple NN architecture was used to develop the prediction system, consisting of an input layer, a hidden layer, and an output layer, as shown in Figure 6. The size of the input layer was equal to the size of the feature vector. The hidden layer consisted of a number of nodes. The output layer consisted of three output nodes as the OST prediction system had three classification groups (short-term, mid-term, and long-term). We used the hyperbolic tangent sigmoid activation function (tanh) in the hidden layer and the softmax activation function in the classification layer. Several configurations were implemented to tune the size of the hidden layer (i.e., the number of NN nodes) for the three sets of features. The best prediction models based on the three approaches are listed in Table 6.

**Figure 7:**
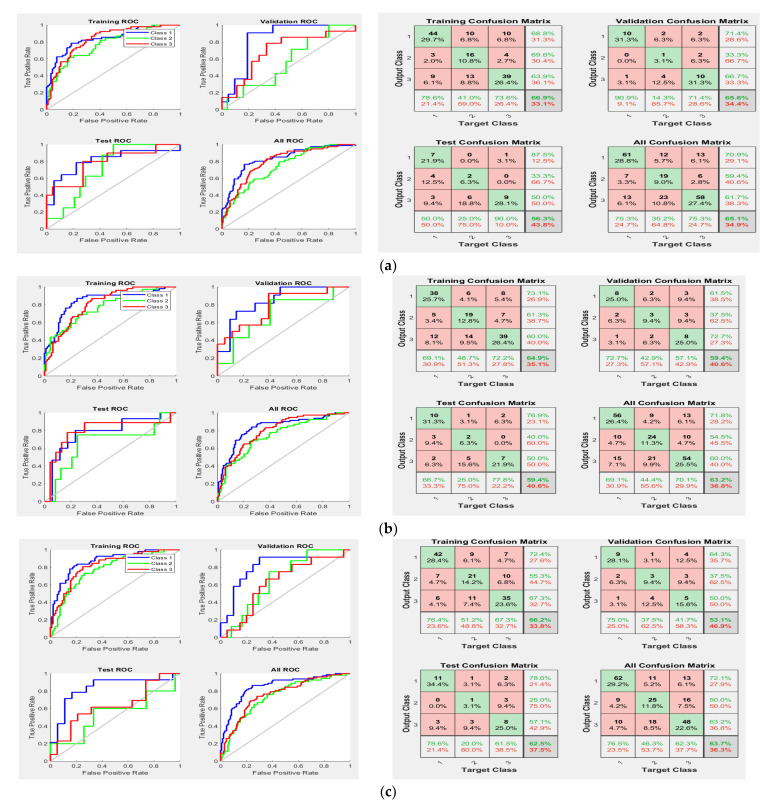
The receiver operating characteristic curves and confusion matrices for models in Experiment 3: (**a**) mid-sagittal approach; (**b**) mid-coronal approach; (**c**) mid-horizontal approach.

For the third experiment, it was found that the surgical treatment improved the survival time for GBM patients [27]; thus, the resection status was added to the three sets of the radiomic features, in addition to the age feature, to improve the performance of the prediction system. Then, the new feature vectors were used to train the NN classifier. Several configurations were implemented to tune the size of the hidden layer, as well as develop the best prediction rate. Table 7 displays the best developed OST models based on feature fusions (radiomic features and non-imaging features) for the three approaches (mid-sagittal, mid-coronal, and mid-horizontal). For better understanding the performance of the developed classification models in Table 7, confusion matrices and receiver operating characteristic curves (ROCs) are presented in Figure 7 for the mid-sagittal, mid-coronal, and mid-horizontal approaches.

## 4. Discussion

The prediction system based on our radiomic feature extraction method and NN is suitable to predict prognosis outcomes for glioblastoma patients by classifying each patient into one of the three survival time groups: short-term survival (<10 months), mid-term survival (10–15 months), and long-term survival (>15 months), as shown in Table 3, Table 4 and Table 5. According to the results in Table 6 and Table 7, the overall accuracy of the OST prediction models based on the three approaches (mid-sagittal, mid-coronal, and mid-horizontal) increased when feature fusions of radiomic and non-imaging features were used to train the NN classifier. As the survival time is better for patients after surgical treatment [27], we expected better improvements in the system performance of Experiment 3 (Table 7) compared to Experiment 2 (Table 6). We believe that these slight improvements of accuracy in the validation and testing datasets of Experiment 3 were due to the unknown resection status for more than half of the samples of the data, which provided unclear descriptions for this feature. We believe that, if we have complete information about the resection status for all of the patients, the test accuracy will increase in Experiment 3.

In addition, according to the results in Figure 7 (confusion matrices and ROCs in the test data), type II errors number of false negatives (FNs)) were more common than type I errors (number of false positive (FPs)) in the mid-term survival (Class 2), but less common than type I errors in the long-term survival (Class 3) for the three approaches of the radiomic features. Type II errors were also more common than type I errors in the short-term survival (Class 1) for both the mid-sagittal and mid-coronal radiomic feature approaches, but they were similar in number to type I errors in the mid-horizontal radiomic feature approach. Moreover, Class 2 had the worst area under the curve (AUC) compared to Class 1 and Class 3 for the three radiomic feature approaches. Thus, the accuracy of Class 2 was the worst compared to Class 1 and Class 3. There are three reasonable reasons for the reduction in accuracy of Class 2. First, the data were unbalanced, as shown in Table 2, whereby the number of patients (i.e., samples) of Class 2 was approximately 30% lower than the number of the patients in Class 1 and Class 3. This could have affected the quality of the developed descriptors, as well as the prediction rate of Class 2. Second, the time period range of Class 2 in months is 6 months (10 months to 15 months) which is small compared to Class 1 (9 months) and Class 3 (more than 15 months, up to 60 months). This might have produced a model with descriptors that contain information from Class 1 and Class 3, which definitely affected the accuracy of Class 2. Third, the survival time of 300 days (10 months) was used to separate Class 2 and Class 1, whereas the survival time of 450 days (15 months) was used to separate Class 2 and Class 3. Thus, Class 2 has two critical zones (one with Class 1 (day 300) and another one with Class 3 (day 450), whereas Class 1 and Class 3 have only one critical zone (day 299) and (day 451) for Class 1 and Class 3, respectively. This allowed Class 2 to have samples containing features of Class 1 and Class 3. To clarify this point, Figure 3b shows there were six patients with an overall survival time of 9 months (short-term), and two of these patients had a survival time of 296 days. Eight patients had an overall survival time of 16 months (long-term), and one of these patients had a survival time of 453 days. This means that, within more or less a few days, these patients would be considered Class 2. Furthermore, one of the patients with a survival time of 10 months (mid-term) had a survival time of 300 days, and two patients with a survival time of 15 months had a survival time of 448 days (mid-term). Thus, these patients were in a critical time interval, which makes them likely to have descriptors from other classes. We believe that increasing size of the training dataset and/or using balanced data can decrease the occurrence of type I errors and type II errors, as well as improve the performance of the prediction system.

Moreover, the test accuracy of the OST prediction model developed in this paper is better than the test accuracy of our previous work, which was based on radiomic features from eight and four brain sub-volumes and shape features [57]. In addition, the results of our prediction system are competitive compared with the top achievements in BraTS 2018–2019 [50,51,52,53,54,55], as their best accuracy in the unseen dataset did not exceed 62%, as shown in Table 1.

## 5. Conclusions and Future Work

Our feature extraction method based on brain functional regions is suitable to develop an automatic OST prediction system for glioblastoma patients based on traditional ML methods, even when a small dataset is used. In addition, the NN showed the ability to develop the best prediction models compared to other ML methods, potentially due to the ability of the NN architecture to render nonlinearity and complexity in a dataset. We planned on testing and evaluating our proposed method in BraTS 2021, but they did not release a survival task this year (2021). To improve the performance of the developed OST prediction system, the following strategies can be used: increasing the size of the dataset, as well as using a balanced dataset by either collecting new samples or using proper data augmentation methods, using completed information of the resection status feature for all patients in the dataset, combining the features of the three proposed approaches, combining other types of radiomic features (shape, texture) with volumetric and location features, and using a multimodal dataset, which contains other types of data that relate to developing aggressive brain tumors, such as genomic data. In addition, as the brain functionality regions (lobes) are responsible for controlling specific organs and functions in a human body, we believe extracting the proposed volumetric features from the brain lobes will improve the performance of the OST prediction system. Moreover, the possibility of developing an accurate OST regression model instead of a classification model can be explored to solve the unbalanced data problem.

## Figures and Tables

**Table 1:** State-of-the-art overall survival time prediction methods for glioblastoma patients using BraTS 2018 and 2019 datasets, where RS refers to the resection status and Acc refers to the accuracy.

Study (Rank-Year)	Features	ML Model	Validation Acc	Test Acc
Feng et al. (1st 2018) [50]	Radiomics (size, shape) + (age, RS)	Linear regression model	32.1%	-
Puybareau et al. (2nd 2018) [51]	Radiomics (size, location) + age	Random forest	54%	61%
Baid et al. (3rd 2018) [52]	Radiomics (shape, statistical, texture) + age	Neural network	57.1%	55.8%
Agravat et al. (1st 2019) [53]	Radiomics (shape, statistical texture) + age	Random forest regressor	58.6%	57.9%
Wang et al. (2nd 2019) [54]	Radiomics (shape, location, texture features) + invasiveness + (age, RS)	Random forest, epsilon-support vector regression	59%	-
Wang et al. (3rd 2019) [55]	Radiomics (size, shape) + age	Fully connected neural network with two hidden layers	44.8%	55.1%

**Table 2:** Characteristics of the survival BraTS 2019 dataset. Resection status (ReS), short-term (StT), mid-term (MdT), long-term (LgT). Notes: the alive sample was discarded from the computations.

Parameter	*n*
Patients	212
Dead patients	211
Alive patients	1
Patients with StT	81
Patients with MdT	55
Patients with LgT	76
Patients with GTR ReS	102
Patients with STR ReS	3
Patients with Unknown ReS	107
Patients with StT in GTR group	35
Patients with MdT in GTR group	27
Patients with LgT in GTR group	39

**Table 3:** Performance of the best OST prediction systems, using radiomic features based on mid-sagittal plane and various ML methods.

No. of Features	ML Method	Overall Accuracy
12	NN (hidden nodes = 150)	53.3%
	SVM (fine Gaussian)	44.3%
	KNN (weighted)	49.5%
	Naïve Bayes (Gaussian)	43.9%
	Linear discriminant	54.8%
	Tree (ensemble)	46%

**Table 4:** Performance of the best OST prediction systems, using radiomic features based on mid-coronal plane and various ML methods.

No. of Features	ML Method	Overall Accuracy
12	NN (hidden nodes = 50)	53.3%
	SVM (fine Gaussian)	45.3%
	KNN (coarse)	44.3%
	Naïve Bayes (Gaussian)	42.5%
	Linear discriminant	43.4%
	Tree (fine)	39.2%

**Table 5:** Performance of the best OST prediction systems, using radiomic features based on mid-horizontal plane and various ML methods.

No. of Features	ML Method	Overall Accuracy
12	NN (hidden nodes = 40)	53.2%
	SVM (fine Gaussian)	48.6%
	KNN (weighted)	49.5%
	Naïve Bayes (Gaussian)	39.6%
	Linear discriminant	42%
	Tree (boosted)	42%

**Table 6:** Performance of the best OST prediction systems, with volumetric and location features and age factor, using NN.

No. of Features	Approach	No. of Nodes	Training Accuracy	Validation Accuracy	Test Accuracy	Overall Accuracy
13	Mid-sagittal	50	62.3%	56.3%	65.6%	63.3%
	Mid-coronal	50	62.2%	56.3%	56.3%	60.4%
	Mid-horizontal	150	68.2%	50%	62.5%	64.6%

**Table 7:** Performance of the best OST prediction systems, with volumetric and location features with age and resection status factors, using NN.

No. of Features	Approach	No. of Nodes	Training Accuracy	Validation Accuracy	Test Accuracy	Overall Accuracy
14	Mid-sagittal	100	66.9%	65.6%	56.3%	65.1%
	Mid-coronal	200	64.9%	59.4%	59.4%	63.2%
	Mid-horizontal	100	66.2%	53.1%	62.5%	63.7%

## Data Availability

The BraTS 2019 dataset was used to develop this work, and it was downloaded from the BraTS challenge after proper registration.

## Appendix A

The type and order of radiomic features extracted based on the three approaches of the proposed method are listed in Table A1, Table A2 and Table A3.

**Table A1:** Volumetric features extracted from mid-sagittal plane approach.

Feature Type	Description
V_wb_	Volume of the whole brain region
V_wt_	Volume of the whole tumor region
V_tL_	Volume of the whole tumor region in the left volume
V_tR_	Volume of the whole tumor region in the right volume
V_bL_	Volume of the brain region in the left volume
V_bR_	Volume of the brain region in the right volume
V_EDL_	Volume of the ED tumor region in the left volume
V_EDR_	Volume of the ED tumor region in the right volume
V_ETL_	Volume of the ET tumor region in the left volume
V_ETR_	Volume of the ET tumor region in the right volume
V_NCRL_	Volume of the NCR tumor region in the left volume
V_NCRR_	Volume of the NCR tumor region in the right volume

**Table A2:** Volumetric features extracted from mid-coronal plane approach.

Feature Type	Description
V_wb_	Volume of the whole brain region
V_wt_	Volume of the whole tumor region
V_tA_	Volume of the whole tumor region in the anterior volume
V_tP_	Volume of the whole tumor region in the posterior volume
V_bA_	Volume of the brain region in the anterior volume
V_bP_	Volume of the brain region in the posterior volume
V_EDA_	Volume of the ED tumor region in the anterior volume
V_EDP_	Volume of the ED tumor region in the posterior volume
V_ETA_	Volume of the ET tumor region in the anterior volume
V_ETP_	Volume of the ET tumor region in the posterior volume
V_NCRA_	Volume of the NCR tumor region in the anterior volume
V_NCRP_	Volume of the NCR tumor region in the posterior volume

**Table A3:** Volumetric features extracted from mid-horizontal plane approach.

Feature Type	Description
V_wb_	Volume of the whole brain region
V_wt_	Volume of the whole tumor region
V_tS_	Volume of the whole tumor region in the superior volume
V_tI_	Volume of the whole tumor region in the inferior volume
V_bS_	Volume of the brain region in the superior volume
V_bI_	Volume of the brain region in the inferior volume
V_EDS_	Volume of the ED tumor region in the superior volume
V_EDI_	Volume of the ED tumor region in the inferior volume
V_ETS_	Volume of the ET tumor region in the superior volume
V_ETI_	Volume of the ET tumor region in the inferior volume
V_NCRS_	Volume of the NCR tumor region in the superior volume
V_NCRI_	Volume of the NCR tumor region in the inferior volume

## References

[1] Ferlay J., Shin H.-R., Bray F., Forman D., Mathers C., Parkin D.M. (2010). Estimates of worldwide burden of cancer in 2008: Globocan 2008. Int. J. Cancer.

[2] Holland E.C. (2001). Progenitor cells and glioma formation. Curr. Opin. Neurol..

[3] Cho H.H., Lee S.H., Kim J., Park H. (2018). Classification of the glioma grading using radiomics analysis. PeerJ.

[4] Louis D.N., Perry A., Reifenberger G., von Deimling A., Figarella-Branger D., Cavenee W.K., Ohgaki H., Wiestler O.D., Kleihues P., Ellison D.W. (2016). The 2016 World Health Organization Classification of Tumors of the Central Nervous System: A summary. Acta Neuropathol..

[5] Claus E.B., Walsh K., Wiencke J.K., Molinaro A.M., Wiemels J.L., Schildkraut J.M., Bondy M.L., Berger M.S., Jenkins R.B., Wrensch M. (2015). Survival and low-grade glioma: The emergence of genetic information. Neurosurg. Focus.

[6] Fisher J.P., Adamson D.C. (2021). Current FDA-Approved Therapies for High-Grade Malignant Gliomas. Biomedicines.

[7] Poon M.T., Sudlow C.L., Figueroa J.D., Brennan P.M. (2020). Longer-term (≥ 2 years) survival in patients with glioblastoma in population-based studies pre- and post-2005: A systematic review and meta-analysis. Sci. Rep..

[8] Hou L.C., Veeravagu A., Hsu A.R., Tse V.C.K. (2006). Recurrent glioblastoma multiforme: A review of natural history and management options. Neurosurg. Focus FOC.

[9] Omerhodžić I., Omerhodžić I., Arnautović K. (2019). Introductory Chapter: Glioma—Merciless Medical Diagnosis. Glioma—Contemporary Diagnostic and Therapeutic Approaches.

[10] Jovčevska I., Kočevar N., Komel R. (2013). Glioma and glioblastoma—How much do we (not) know?. Mol. Clin. Oncol..

[11] Tamim A.F., Juweid M., De Vleeschouwer S. (2017). Epidemiology and Outcome of Glioblastoma. Glioblastoma.

[12] Lorenzo M.F., Arena C.B., Davalos R.V., De Vleeschouwer S. (2017). Maximizing Local Access to Therapeutic Deliveries in Glioblastoma. Part III: Irreversible Electroporation and High-Frequency Irreversible Electroporation for the Eradication of Glioblastoma. Glioblastoma.

[13] Ohgaki H., Kleihues P. (2013). The definition of primary and secondary glioblastoma. Clin. Cancer Res..

[14] Dorsey J.F., Salinas R.D., Dang M., Alonso-Basanta M., Judy K.D., Maity A., Lustig R.A., Lee J.Y.K., Philips P.C., Pruitt A., Niederhuber J.E., Armitage J.O., Doroshow J.H., Kastan M.B., Tepper J.E. (2020). Chapter 63: Cancer of the central nervous system. Abeloff’s Clinical Oncology.

[15] Shu X.-O., Jin F., Linet M.S., Zheng W., Clemens J., Mills J., Gao Y.-T. (1994). Diagnostic X-ray and ultrasound exposure and risk of childhood cancer. Br. J. Cancer.

[16] Jiang H., Cui Y., Wang J., Lin S. (2017). Impact of epidemiological characteristics of supratentorial gliomas in adults brought about by the 2016 world health organization classification of tumors of the central nervous system. Oncotarget.

[17] Ostrom Q.T., Cioffi G., Gittleman H., Patil N., Waite K., Kruchko C., Barnholtz-Sloan J.S. (2019). CBTRUS Statistical Report: Primary Brain and Other Central Nervous System Tumors Diagnosed in the United States in 2012-2016. Neuro Oncol..

[18] Persaud-Sharma D., Burns J., Trangle J., Moulik S. (2017). Disparities in Brain Cancer in the United States: A Literature Review of Gliomas. Med. Sci..

[19] Dorsey J.F., Hollander A.B., Alonso-Basanta M., Macyszyn L., Bohman L.E., Judy K.D., Maity A., Lee J.Y.K., Lustig R.A., Philips P.C., John E.N., James O.A., James H.D., Michael B.K., Joel E.T. (2014). 66—Cancer of the Central Nervous System. Abeloff’s Clinical Oncology.

[20] Hanif F., Muzaffar K., Perveen K., Malhi S.M., Simjee S.U. (2017). Glioblastoma Multiforme: A Review of its Epidemiology and Pathogenesis through Clinical Presentation and Treatment. Asian Pac. J. Cancer Prev..

[21] Tandel G.S., Biswas M., Kakde O.G., Tiwari A., Suri H.S., Turk M., Laird J.R., Asare C.K., Ankrah A.A., Khanna N.N. (2019). A Review on a Deep Learning Perspective in Brain Cancer Classification. Cancers.

[22] Nelson S.J., Cha S. (2003). Imaging Glioblastoma Multiforme. Cancer J..

[23] Menze B.H., Jakab A., Bauer S., Kalpathy-Cramer J., Farahani K., Kirby J., Burren Y., Porz N., Slotboom J., Wiest R. (2015). The Multimodal Brain Tumor Image Segmentation Benchmark (BRATS). IEEE Trans. Med. Imaging.

[24] D’Alessio A., Proietti G., Sica G., Scicchitano B.M. (2019). Pathological and Molecular Features of Glioblastoma and Its Peritumoral Tissue. Cancers.

[25] Kazerooni A.F., Nabil M., Zadeh M.Z., Firouznia K., Azmoudeh-Ardalan F., Frangi A.F., Davatzikos C., Rad H.S. (2018). Characterization of active and infiltrative tumorous subregions from normal tissue in brain gliomas using multiparametric MRI. J. Magn. Reson. Imaging.

[26] Wu C.X., Lin G.S., Lin Z.X., Zhang J.D., Liu S.Y., Zhou C.F. (2015). Peritumoral edema shown by MRI predicts poor clinical outcome in glioblastoma. World J. Surg. Oncol..

[27] Qin X., Liu R., Akter F., Qin L., Xie Q., Li Y., Qiao H., Zhao W., Jian Z., Liu R. (2021). Peri-tumoral brain edema associated with glioblastoma correlates with tumor recurrence. J. Cancer.

[28] Kumar S., Handa A., Sinha R., Tiwari R. (2013). Bilateral cystic glioblastoma multiforme. J. Neurosci. Rural. Pract..

[29] Baid U., Ghodasara S., Mohan S. (2021). The RSNA-ASNR-MICCAI BraTS 2021 Benchmark on Brain Tumor Segmentation and Radiogenomic Classification. arXiv.

[30] Seidel C., Dörner N., Osswald M., Wick A., Platten M., Bendszus M., Wick W. (2011). Does age matter?—A MRI study on peritumoral edema in newly diagnosed primary glioblastoma. BMC Cancer.

[31] Carlson M.R., Pope W.B., Horvath S., Braunstein J.G., Nghiemphu P., Tso C.L., Mellinghoff I., Lai A., Liau L.M., Mischel P.S. (2007). Relationship between Survival and Edema in Malignant Gliomas: Role of Vascular Endothelial Growth Factor and Neuronal Pentraxin 2. Clin. Cancer Res..

[32] Choi S.J., Hwang H.Y., Kim N.R., Lee S.-W., Kim J.H., Choi H.-Y., Kim H.-S. (2010). The Radiologic Features of Cystic versus Noncystic Glioblastoma Multiforme as Significant Prognostic Factors. J. Korean Soc. Radiol..

[33] Yee P.P., Wei Y., Kim S.-Y., Lu T., Chih S.Y., Lawson C., Tang M., Liu Z., Anderson B., Thamburaj K. (2020). Neutrophil-induced ferroptosis promotes tumor necrosis in glioblastoma progression. Nat. Commun..

[34] Berhouma M., Jacquesson T., Jouanneau E., Cotton F. (2019). Pathogenesis of peri-tumoral edema in intracranial meningiomas. Neurosurg. Rev..

[35] Shukla G., Alexander G.S., Bakas S., Nikam R., Talekar K., Palmer J.D., Shi W. (2017). Advanced magnetic resonance imaging in glioblastoma: A review. Chin. Clin. Oncol..

[36] Akbari H., Macyszyn L., Da X., Wolf R.L., Bilello M., Verma R., O’Rourke D., Davatzikos C. (2014). Pattern analysis of dynamic susceptibility contrast-enhanced MR imaging demonstrates peritumoral tissue heterogeneity. Radiology.

[37] Schoenegger K., Oberndorfer S., Wuschitz B., Struhal W., Hainfellner J., Prayer D., Heinzl H., Lahrmann H., Marosi C., Grisold W. (2009). Peritumoral edema on MRI at initial diagnosis: An independent prognostic factor for glioblastoma?. Eur. J. Neurol..

[38] Ding X., Shang B. (2021). DeepBAR: A Fast and Exact Method for Binding Free Energy Computation. J. Phys. Chem. Lett..

[39] Jeon J., Nim S., Teyra J., Datti A., Wrana J.L., Sidhu S.S., Moffat J., Kim P.M. (2014). A systematic approach to identify novel cancer drug targets using machine learning, inhibitor design and high-throughput screening. Genome Med..

[40] Jeon J., Nim S., Teyra J., Datti A., Wrana J.L., Sidhu S.S., Moffat J., Kim P.M. (2016). Automatic planning of head and neck treatment plans. J. Appl. Clin. Med. Phys..

[41] Shiraishi S., Moore K.L. (2016). Knowledge-based prediction of three-dimensional dose distributions for external beam radiotherapy. Med. Phys..

[42] Monz M., Küfer K.H., Bortfeld T.R., Thieke C. (2008). Pareto navigation—Algorithmic foundation of interactive multi-criteria IMRT planning. Phys. Med. Biol..

[43] McIntosh C., Conroy L., Tjong M.C., Craig T., Bayley A., Catton C., Gospodarowicz M., Helou J., Isfahanian N., Kong V. (2021). Clinical integration of machine learning for curative-intent radiation treatment of patients with prostate cancer. Nat. Med..

[44] Attiyeh M.A., Chakraborty J., Doussot A., Ba L.L.-E., Mainarich S., Gönen M., Balachandran V.P., D’Angelica M.I., DeMatteo R.P., Jarnagin W.R. (2018). Survival Prediction in Pancreatic Ductal Adenocarcinoma by Quantitative Computed Tomography Image Analysis. Ann. Surg. Oncol..

[45] Priya S., Agarwal A., Ward C., Locke T., Monga V., Bathla G. (2021). Survival prediction in glioblastoma on post-contrast magnetic resonance imaging using filtration based first-order texture analysis: Comparison of multiple machine learning models. Neuroradiol. J..

[46] Zhang Y., Lobo-Mueller E.M., Karanicolas P., Gallinger S., Haider M.A., Khalvati F. (2020). CNN-based survival model for pancreatic ductal adenocarcinoma in medical imaging. BMC Med. Imaging.

[47] Haarburger C., Weitz P., Rippel O., Merhof D. Image-Based Survival Prediction for Lung Cancer Patients Using CNNS. Proceedings of the 2019 IEEE 16th International Symposium on Biomedical Imaging (ISBI 2019).

[48] Asenjo J., Solís M.L.A., Crimi A., Bakas S. (2021). MRI Brain Tumor Segmentation Using a 2D-3D U-Net Ensemble. Brainlesion: Glioma, Multiple Sclerosis, Stroke and Traumatic Brain Injuries.

[49] Liu Z., Zhu G., Jiang X., Zhao Y., Zeng H., Jing J., Ma X. (2020). Survival Prediction in Gallbladder Cancer Using CT Based Machine Learning. Front Oncol..

[50] Feng X., Tustison N., Meyer C., Crimi A., Bakas S., Kuijf H., Keyvan F., Reyes M., van Walsum T. (2019). Brain Tumor Segmentation Using an Ensemble of 3D U-Nets and Overall Survival Prediction Using Radiomic Features. Brainlesion: Glioma, Multiple Sclerosis, Stroke and Traumatic Brain Injuries.

[51] Puybareau E., Tochon G., Chazalon J., Fabrizio J., Crimi A., Bakas S., Kuijf H., Keyvan F., Reyes M., van Walsum T. (2019). Segmentation of Gliomas and Prediction of Patient Overall Survival: A Simple and Fast Procedure. Brainlesion: Glioma, Multiple Sclerosis, Stroke and Traumatic Brain Injuries.

[52] Baid U., Talbar S.N., Rane S.U., Gupta S., Thakur M.H., Moiyadi A., Thakur S., Mahajan A., Crimi A., Bakas S., Kuijf H., Keyvan F., Reyes M., van Walsum T. (2018). Deep Learning Radiomics Algorithm for Gliomas (DRAG) Model: A Novel Approach Using 3D UNET Based Deep Convolutional Neural Network for Predicting Survival in Gliomas. Brainlesion: Glioma, Multiple Sclerosis, Stroke and Traumatic Brain Injuries.

[53] Agravat R.R., Raval M.S., Crimi A., Bakas S. (2020). Brain Tumor Segmentation and Survival Prediction. Brainlesion: Glioma, Multiple Sclerosis, Stroke and Traumatic Brain Injuries.

[54] Wang S., Dai C., Mo Y., Angelini E., Guo Y., Bai W., Crimi A., Bakas S. (2020). Automatic Brain Tumour Segmentation and Biophysics-Guided Survival Prediction. Brainlesion: Glioma, Multiple Sclerosis, Stroke and Traumatic Brain Injuries.

[55] Wang F., Jiang R., Zheng L., Meng C., Biswal B., Crimi A., Bakas S. (2020). 3D U-Net Based Brain Tumor Segmentation and Survival Days Prediction. Brainlesion: Glioma, Multiple Sclerosis, Stroke and Traumatic Brain Injuries.

[56] Multimodal Brain Tumor Segmentation Challenge 2019: Data. https://www.med.upenn.edu/cbica/brats2019/data.html.

[57] Chato L., Kachroo P., Latifi S., Crimi A., Bakas S. (2021). An Automatic Overall Survival Time Prediction System for Glioma Brain Tumor Patients Based on Volumetric and Shape Features. Brainlesion: Glioma, Multiple Sclerosis, Stroke and Traumatic Brain Injuries.

